# Audible changes in marine trophic ecology: Baleen whale song tracks foraging conditions in the eastern North Pacific

**DOI:** 10.1371/journal.pone.0318624

**Published:** 2025-02-26

**Authors:** John P. Ryan, William K. Oestreich, Kelly J. Benoit-Bird, Chad M. Waluk, Carlos A. Rueda, Danelle E. Cline, Yanwu Zhang, Ted Cheeseman, John Calambokidis, James A. Fahlbusch, Jack Barkowski, Alyson H. Fleming, Calandra N. Turner Tomaszewicz, Jarrod A. Santora, Tetyana Margolina, John E. Joseph, Ari S. Friedlaender, Jeremy A. Goldbogen

**Affiliations:** 1 Research Department, Monterey Bay Aquarium Research Institute, Moss Landing, California, United States of America; 2 Engineering Department, Monterey Bay Aquarium Research Institute, Moss Landing, California, United States of America; 3 Marine Ecological Research Centre, Southern Cross University, Lismore, New South Wales, Australia; 4 Happywhale.com, Santa Cruz, California, United States of America; 5 Cascadia Research Collective, Olympia, Washington, United States of America; 6 Hopkins Marine Station, Stanford University, Pacific Grove, California, United States of America; 7 Nelson Institute for Environmental Studies, University of Wisconsin, Madison, Wisconsin, United States of America; 8 Marine Mammal and Turtle Division, NOAA Southwest Fisheries Science Center, La Jolla, California, United States of America; 9 Department of Applied Math, University of California, Santa Cruz, California, United States of America; 10 Fisheries Ecology Division, Southwest Fisheries Science Center, National Marine Fisheries Service, NOAA, Santa Cruz, California, United States of America; 11 Oceanography Department, Naval Postgraduate School, Monterey, California, United States of America; 12 Institute of Marine Sciences, University of California, Santa Cruz, California, United States of America; Wildlife Conservation Society Canada, CANADA

## Abstract

Among tremendous biodiversity within the California Current Ecosystem (CCE) are gigantic mysticetes (baleen whales) that produce structured sequences of sound described as song. From six years of passive acoustic monitoring within the central CCE we measured seasonal and interannual variations in the occurrence of blue (*Balaenoptera musculus*), fin (*Balaenoptera physalus*), and humpback (*Megaptera novaeangliae*) whale song. Song detection during 11 months of the year defines its prevalence in this foraging habitat and its potential use in behavioral ecology research. Large interannual changes in song occurrence within and between species motivates examination of causality. Humpback whales uniquely exhibited continuous interannual increases, rising from 34% to 76% of days over six years, and we examine multiple hypotheses to explain this exceptional trend. Potential influences of physical factors on detectability – including masking and acoustic propagation – were not supported by analysis of wind data or modeling of acoustic transmission loss. Potential influences of changes in local population abundance, site fidelity, or migration timing were supported for two of the interannual increases in song detection, based on extensive local photo ID data (17,356 IDs of 2,407 individuals). Potential influences of changes in foraging ecology and efficiency were supported across all years by analyses of the abundance and composition of forage species. Following detrimental food web impacts of a major marine heatwave that peaked during the first year of the study, foraging conditions consistently improved for humpback whales in the context of their exceptional prey-switching capacity. Stable isotope data from humpback and blue whale biopsy samples are consistent with observed interannual variations in the regional abundance and composition of forage species. This study thus indicates that major interannual changes in detection of baleen whale song may reflect underlying variations in forage species availability driven by energetic variations in ecosystem state.

## Introduction

Monitoring dynamic marine ecosystems for improved ecological understanding and better-informed resource management is a globally important challenge. In recent decades, major advances in remote sensing technologies – including satellites [1], coastal radar stations [2], autonomous platforms [3–5], and uncrewed aerial systems [6] – have contributed to the ability to observe physical, chemical, and biological processes in marine ecosystems with increasing precision and detail. Despite these advances, monitoring species of higher trophic levels at ecosystem scales remains a significant challenge. Remote sensing via passive acoustic monitoring (PAM) is essential for filling this gap for several reasons. Most notably, the aquatic medium and limited visibility in marine ecosystems has led to the evolution of long-distance acoustic communication in marine animals [7], allowing for wide-ranging detection of soniferous species and their behavior using hydrophones [8]. In this study, we examine how variations in detection of song from three baleen whale (*Mysticeti)* species with distinct foraging strategies inhabiting the central California Current Ecosystem (CCE) – blue whales (*B. m. musculus musculus*), fin whales (*B. p. physalus*), and humpback whales (*M. n. novaeangliae*) – track variations in ecosystem state and forage species abundance and composition.

**Fig 1:**
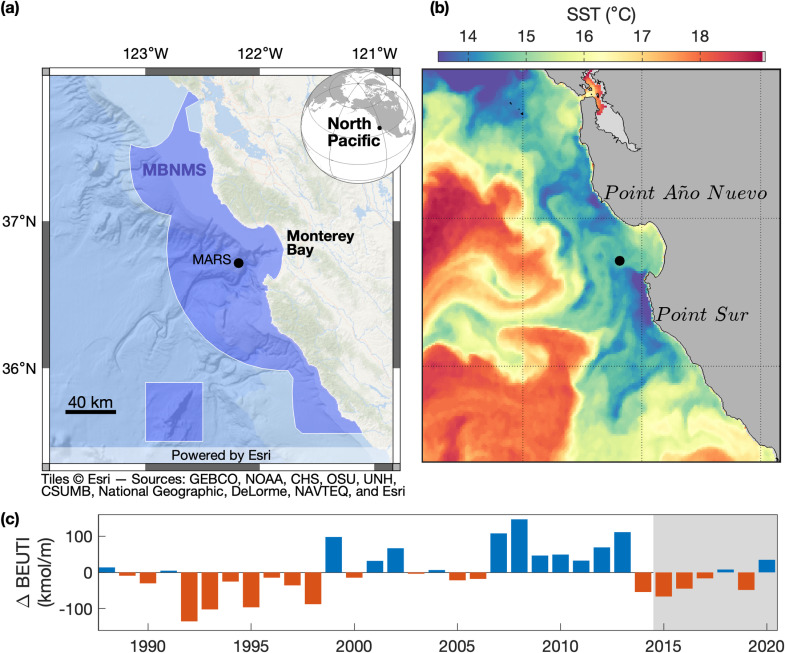
Ecosystem setting. (a) The passive acoustic monitoring site is in Monterey Bay National Marine Sanctuary (MBNMS) along the eastern margin of the North Pacific. Hydrophones are operated through the Monterey Accelerated Research System (MARS) cabled observatory (main node at 36.713°N, 122.186°W, 891 m depth). The ocean base map is the intellectual property of Esri and is used herein under license. Copyright © Esri. All rights reserved. (b) A sea surface temperature (SST) image acquired on 13 October 2020 shows the process that fuels primary productivity, upwelling of deep nutrient-rich water. Upwelling is illustrated by filamentous cool SST plumes that are coldest where they originate along the coast, which tends to be around Point Año Nuevo and Point Sur in the Monterey Bay region [31]. (c) Annual biologically effective upwelling transport index (BEUTI) at 37°N represents the vertical flux of nitrate into the surface mixed layer per meter of coastline [17]. To emphasize interannual variation, BEUTI is presented as annual differences from the long term mean of annual totals (174 kmol/ m). The shaded period identifies the years for which acoustic detection of baleen whale species is examined.

Blue, fin, and humpback whale populations all exploit the tremendous seasonal biological productivity of the CCE to support their large body size and long-distance movements [9,10]. This eastern boundary upwelling ecosystem also supports diverse and abundant populations of other ecologically, economically, and culturally significant organisms. Upwelling of deep, nutrient-rich water in the CCE (Fig 1) results from seasonal equatorward wind forcing and supports the growth of phytoplankton [11,12]. This primary productivity propagates to higher trophic levels, sustaining seasonally abundant forage species on which numerous and diverse predators depend [13]. In addition to strong seasonality in physical and biological oceanographic conditions, this upwelling system is marked by significant interannual variability in upwelling magnitude and phenology, levels of primary productivity, and relative abundance of distinct forage species [14–19]. For example, a persistent marine heatwave during 2014–2016 changed regional upwelling dynamics and phytoplankton ecology, in turn impacting the behavior and population health of invertebrate and vertebrate animal species [20–24]. At decadal time scales, large-scale physical oscillations that drive changes in forage species abundance and composition are evident in the diets of humpback whales, as revealed by stable isotope analysis [25,26]. These impacts at higher trophic levels suggest that top predator populations could act as ecosystem sentinels [27]. Some predator populations have recently been assessed in this context, e.g., seabirds [28], humpback whales [29] and blue whales [30], each providing insights into marine heatwave impacts on forage species and predator ecology.

Blue, fin, and humpback whales exhibit different foraging strategies and seasonality of presence in this ecosystem. Eastern North Pacific blue whales are obligate euphausiid (krill) predators which typically forage along the shelf break off western North America during the summer and fall before migrating southward to lower-latitude breeding grounds [32,33]. The timing of blue whale arrival and departure from CCE foraging habitat can vary by several months interannually in association with changes in the phenology of physical and biological oceanographic processes which drive krill abundance, density, and distribution [19,34]. Fin whales in the eastern North Pacific also prey primarily on euphausiids, although copepods may contribute substantially to their diet in some years [35]. Studies employing biotelemetry [36] and PAM [37] suggest that fin whales occupy the CCE year-round. Tag deployments on fin whales in the Southern California Bight indicated two seasonal patterns: (1) migration of part of the population to lower latitudes in winter, and (2) preferential habitat occupancy nearshore during fall and winter and offshore during spring and summer [36]. Relatively little is known about the habitat preferences and seasonality of fin whales in the central CCE. Humpback whales that forage in the central CCE belong almost exclusively to two of the distinct population segments in the northeastern Pacific, migrating between foraging grounds off the western United States and breeding grounds off Mexico and Central America [38–41]. While migration between foraging grounds and lower latitude breeding grounds is well documented, visual sighting and PAM data indicate nearly year-round presence of humpback whales in the central CCE [29]. During their time in foraging habitat off western North America, humpback whales target both krill and forage fish (anchovy and sardine), flexibly switching between dominant prey sources depending on oceanographic conditions [26].

**Fig 2:**
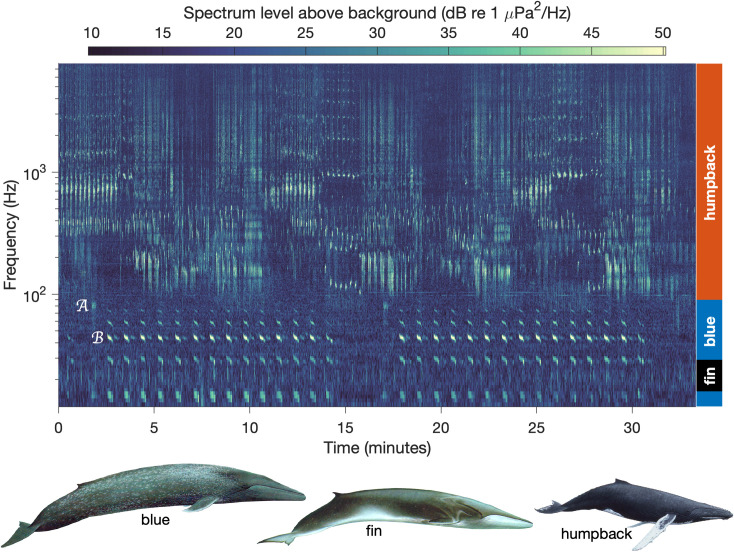
Mysticete symphony. The spectrogram shows simultaneous song (structured sound sequences) from three baleen whale species — blue, fin and humpback — recorded through the MARS cabled observatory (Fig 1) on 1 January 2018. The approximate frequency range used by each species is indicated at the right. One blue whale A and B call each is labeled immediately before an example; the B call is labeled at the third and most energetic harmonic. Spectrogram parameters: sample frequency fs = 16 kHz, length of fast Fourier transform (FFT) for each segment nfft = 16,000, Hanning window, 50% overlap between segments. The frequency dependent first percentile of spectrum levels was subtracted to emphasize song signal above a frequency-dependent background. Whale artist: Larry Foster.

Singing behavior of blue, fin and humpback whales, involving production of sound sequences that are rhythmic and structured, is thought to be limited to mature males [42–45]. While song is often associated with reproductive behavior [42,45,46], song detection has also been used to derive insights into foraging dynamics [30,47,48], migration timing [19,49], cultural transmission of behavior [50], trends in relative abundance and patterns of spatial distribution [51,52]. This information-rich acoustic signal (Fig 2) can be detected by a single hydrophone over an area covering thousands of km^2^ [29,49], and it can dominate marine soundscape spectra [53–56], making acoustic monitoring of baleen whale song an effective remote sensing tool of large marine ecosystems [57,58].

Central to the context of this study is that singing occurs extensively within foraging habitat of the central CCE. Previous studies in this region have detected song from blue whales 7 months of the year and from humpback whales 9 months of the year [29,49]. Song detection in both species can begin months before the winter breeding migration. Humpback whale song can persist for months following the breeding season, after whales return to foraging habitat in spring, a pattern also observed in foraging habitat of the western North Atlantic [59]. In Antarctic foraging habitat, humpback whale song can have the level of complexity found in breeding habitat, and it has been detected to occur during foraging dives [60]. A growing body of literature is recognizing the prevalence of this behavior within foraging habitat, in addition to migratory [61–63] and breeding habitat [51,52].

Detection of both song (Fig 2) and foraging-associated calls can contribute to ecological research. Foraging-associated calls are produced by blue and fin whales [44,64,65], however they are much less prevalent than song in our study region. Blue whale tag data from the eastern North Pacific show that rates of song calls can exceed those of foraging-associated calls by an order of magnitude [44]. PAM data for fin whales off Southern California show a greater proportion of hours per week and a greater proportion of the year during which song calls (20 Hz pulses) were detected compared to foraging-associated calls (40 Hz pulses) [64]. No studies have identified foraging-specific humpback whale calls in our study region. The lesser occurrence of foraging associated calls and the inability to consistently examine their occurrence across all three whale species motivate the focus of the present study on detection of song, which is a more abundant signal that can be described across all three whale species.

Here we combine measured detection of blue, fin, and humpback whale song with concurrent data on whale photo ID, forage species abundance and behavior, isotope-based diet analysis from whale skin biopsy samples, oceanographic and meteorological conditions, and modeling of acoustic propagation to derive insights into the presence and foraging ecology of these sympatric mysticetes. We apply these methods within a six-year study period, July 2015 through June 2021, in the central CCE to address two questions: (1) How does song detection vary seasonally and interannually? (2) Are interannual variations in detection of song indicative of ecosystem state and availability of key forage species? In answering these questions, we show how the songs produced by these gigantic vertebrates can reveal complex dimensions of their ecology in a vast and highly variable ecosystem.

## Materials and methods

### Acoustic recordings

Acoustic recordings were acquired through the Monterey Accelerated Research System (MARS) cabled observatory, located on the continental slope outside Monterey Bay, California (Fig 1a). Since July 2015, MARS has supported nearly continuous recording at a sample rate of 256 kHz using an Ocean Sonics icListen HF, an omnidirectional hydrophone with a bandwidth of 10 Hz to 200 kHz. The data are streamed to a computer on shore and stored in 10-minute wav files. Two derived data products supported analyses: (1) daily files of original audio decimated to a 16 kHz sample rate [66], which enabled humpback whale song detection, and (2) daily files of power spectra with temporal and frequency resolutions of 5 seconds and 1 Hz, respectively, which enabled blue and fin whale song detection. Daily acoustic metrics, described below, were the basis for examination of seasonal and interannual patterns. Only days with at least 75% recording coverage were included in analyses. Of 2192 days within the 6-year period, 96% (2111) were sampled, 97% of sampled days (2051) had coverage of 75% or greater, and 92% of sampled days (1948) had complete coverage.

### Detecting whale song and quantifying its variations

Detection of humpback whale song (Fig 2) employed a deep convolutional neural network (CNN) that was previously trained based on recordings of North Pacific humpback whale song and proven to generalize well across a range of recording and noise conditions [67]. The model frames input audio into analysis windows and produces a corresponding sequence of logistic units as scoring of humpback whale song detection, ranging between 0 and 1. We resampled input data to 10 kHz, as used in the original model development, and applied the CNN model to obtain scores at 1-second resolution over the 6-year study period. To validate model application to MARS recordings, we manually examined two years of results by directly comparing CNN scores with temporally aligned high-resolution spectrograms [58]. This comparison confirmed reliable and consistent detection of humpback whale song, even amid substantial background noise, and it was used to establish the threshold score used for classification. Because two other biological sound sources, gray whales and dolphins, at times produced moderately elevated scores (~0.3 to 0.6), we applied a minimum score threshold of 0.7 to reliably detect humpback whale song and distinguish it from other signals. For each day we quantified the percent of recording time during which the score exceeded this threshold. A minimum daily value of 3% of recording time with a score exceeding 0.7 was applied to define daily presence of song. Performance metrics based on these criteria are summarized in the results.

**Fig 3:**
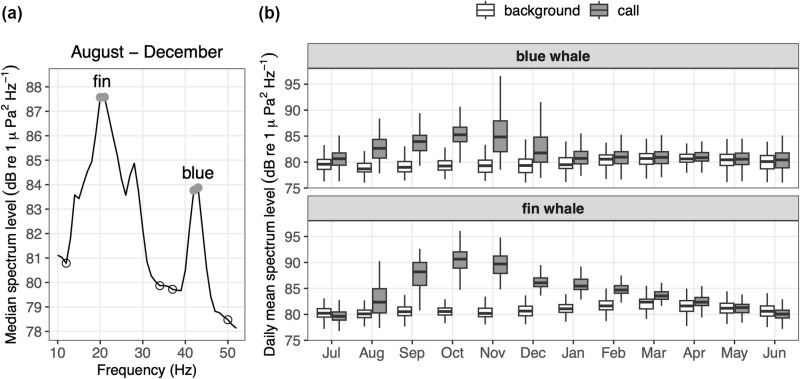
Call index methods for blue and fin whale acoustic detection. (a) The long-term median of daily mean spectrum levels measured at MARS (Fig 1) for the months of August through December shows peaks caused by fin whale 20 Hz pulses and the third harmonic of blue whale B calls (Fig 2). The frequencies at which spectrum levels were used to compute the call index are indicated: peak call energy (solid gray circles) and adjacent background (open circles, one 1-Hz band above and below each peak). (b) Monthly boxplot of daily mean spectrum levels, based on the full time-series, illustrate seasonal changes at the peak frequencies of blue and fin whale calls relative to background. Shown are the interquartile range (IQR, box), median (thick horizontal line), minimum (lower limit of vertical line), and the third quartile + 1.5 × IQR (upper limit of vertical line).

Detection of blue and fin whale song (Fig 2) used energy detection methods that focus on the most prevalent calls produced by these species in the study region: the fin whale 20 Hz call and the blue whale B call. This prevalence is indicated by previous studies in the CCE [44,64] as well as visual examination of high-resolution spectrograms from our monitoring site across all years of the study [68]. For each species we computed a call index (CI; [37,49,69]) as the ratio of the peak spectrum level within the frequency band of the call to the average spectrum level in adjacent frequency bands that are uninfluenced by low-frequency baleen whale vocalizations. CI was calculated at daily resolution using daily mean calibrated power spectra. This method has two advantages: (1) it quantifies the integrated energy of calling, regardless of whether or not chorusing occurs, and (2) it minimizes potential bias from ephemeral noise sources affecting background spectrum levels. The biophonic signal underlying this method is represented by mean spectrum levels across the frequency range spanning both call types (Fig 3a) and the statistical distributions of daily mean spectrum levels in peak and background bands (Fig 3b). The ability to quantify sound energy of targeted whale calls using this method is illustrated by the relative stability in background frequency bands compared to strong seasonal elevation of spectrum levels in the frequency range of the fin whale 20 Hz call (peak 20–21 Hz) and the third harmonic of the blue whale B call (peak 42–43 Hz). For both blue and fin whales, a minimum CI value of 1.01 was applied to define song presence. Considering that CI = 1.0 represents no signal and maximum daily CI values are approximately 1.2 for both species, the minimum threshold of 1.01 represents a signal approximately 5% above the floor of the dynamic range.

To qualify the daily blue and fin whale energy detection metrics as representative of song, additional analyses were conducted. For blue whales we examined occurrence of a different call produced in songs: the A call [70]. In the blue whale song represented in Fig 2, two A calls are represented (label A next to the first). Each A call was followed by a series of B calls (label B next to the third harmonic of one B call; 14 and 15 repetitions, respectively). Considering the core daily detection metric for B calls, we manually examined high-resolution spectrograms for every day within the 6-year study period for which the B-call metric exceeded the minimum threshold, and we quantified cooccurrence of A and B calls. For fin whales we also examined high-resolution spectrograms throughout the time-series to determine whether fin whale 20 Hz calls consistently occurred in series with regular interpulse intervals that are characteristic of song [71].

Daily time-series of metrics for all species were smoothed with a 5-day running mean to clarify temporal boundaries of persistent song presence/ absence while retaining clear description of variations within the annual period of song occurrence. The smoothed, quality-controlled daily time-series comprised the basis for examination of both seasonal and interannual patterns in acoustic detection. Considering the different methods of detecting and quantifying song occurrence across species, it is essential to apply a single consistent metric of seasonal and interannual detection levels. The metric we apply is the percent of recorded days during which detection metrics exceeded their minimum thresholds. For the seasonal time scale, we combined data from all years to define a monthly climatology of the percent of days with detection. For the interannual time scale, we computed the percent of days with detection in each year. Because of the seasonal pattern of baleen whale song, which arises in all three species between July (blue) and August (fin, humpback), each analysis year spanned July 1 of a calendar year through June 30 of the following calendar year.

Previous studies of blue whale song demonstrated that the trend in the day:night ratio of song detection during fall-winter can reveal the timing of the southward breeding migration, which covaried with upwelling and differed by as long as 4 months among the years studied [19,49]. This metric is thus included in our study as a potential indicator of behavioral ecology across all whale species examined. It is computed from the full-resolution song metrics (1 second for humpback whale song scores, 5 seconds for blue and fin whale call indices) binned into solar elevation (SE) ranges that define day (SE > 0°) and night (SE < −12°).

### Examining causality of interannual variations in song detection

Using interdisciplinary observations and modeling, we consider multiple hypotheses to explain causality of the major patterns of interannual variation in detection of whale song. These hypotheses involve ecological variables that affect the source signal within the domain of acoustic reception, and environmental variables that affect signal transmission to the receiver and signal distinction within the greater soundscape. Behavioral ecology variables include whale local abundance, proximity to MARS, site fidelity, migration timing, and the acoustic behavior of mature male (singing) individuals. Environmental variables include physical conditions that determine acoustic propagation, and noise sources that can mask song signal and inhibit detection. Hypotheses are not mutually exclusive, they cannot be completely examined for any of the whale species, and they cannot all be directly examined from available data. However, hypotheses can be most fully considered for humpback whales because extensive photo ID data are uniquely available for this species. These data enable quantitative metrics of local abundance, site fidelity, and migration timing. Below we summarize the ancillary data and modeling resources applied.

#### Whale sightings and photo identification.

**Fig 4:**
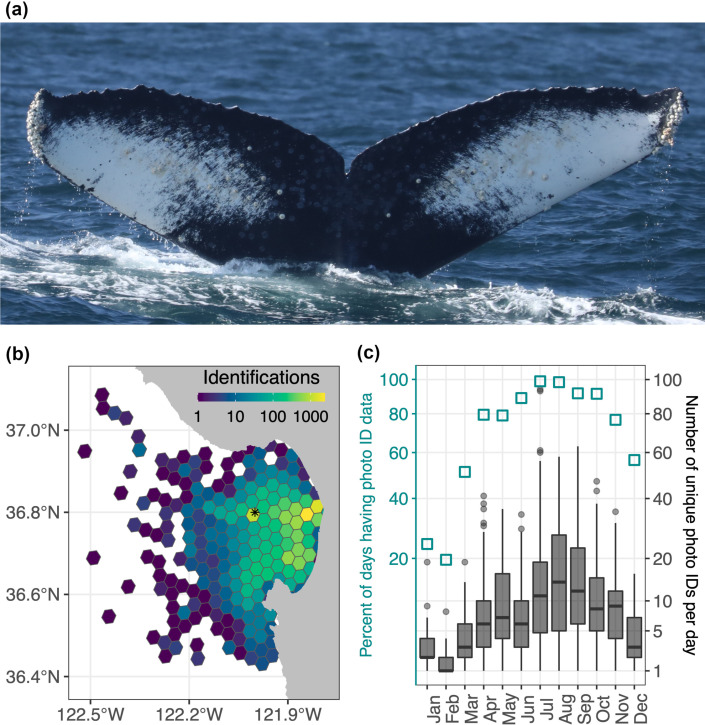
Humpback whale photo ID data and analysis. (a) Example photo of a humpback whale fluke from which identification of individuals is enabled through distinction of fluke shape and coloration. This photo by T. Cheeseman is of the individual most frequently identified in the Monterey Bay region during the study period, *Fran*, who was killed by a ship strike in August 2022. (b) Spatial distribution of *Happywhale* photo ID data over the 6-year study period (n = 17,356); ID counts were summed within equal area grid cells of 10.7 km^2^ using the R package dggridR. Photos from Monterey Bay without position metadata (5.8% of records) were assigned to 36.8°N, 122°W (marked by asterisk). (c) Monthly summary for the six-year study period: percent of days having photo ID data (left axis) and a boxplot of the base visual detection metric, the number of unique individuals identified per day (right axis). Shown are the interquartile range (IQR, box), median (thick horizontal line), minimum (lower limit of vertical line), the third quartile + 1.5 × IQR (upper limit of vertical line), and outliers (gray circles).

To examine seasonal and interannual variation in visual detection in relation to acoustic detection, we utilized identifications of individual whales collected by researchers, naturalists, and citizen scientists aided by a convolutional neural network-based photo ID algorithm [72]. Of the three whale species studied, only humpback whales persistently occupy habitat within the typical domain of whale-watch tours and associated photographic sampling, and CNN application to humpback fluke photos (Fig 4a) is fully developed, so application is constrained to this species. The photo archive came from photographs contributed through a web-based marine mammal photo ID collaboration system, *Happywhale*, from which we examined a subset of a North Pacific-wide dataset [73]. Compared to simple encounter counts from sighting data, using photo ID to quantify the number of unique individuals and their persistence in the recording area enhances comparison with acoustic detection metrics. For our 6-year study period of July 2015 through June 2021, 17,356 identifications (Fig 4b) of 2407 unique individuals were used to compute a base statistic: the number of unique individuals identified each day (UID/day). The value of frequent sampling is evident in the percentage of days that have photographic ID data (Fig 4c).

From the daily UID time-series we computed three visual detection metrics that are relevant to acoustic detection. The first metric, *local population abundance*, is based on analysis of UID/day at seasonal and interannual time scales. Seasonal variation was examined at monthly resolution within individual years and climatologically (pooling UID/day data monthly across all years), and interannual variation was examined from UID/day data pooled within each study year. Comparisons applied graphical (boxplot) representation and evaluation of significant differences using paired Wilcoxon rank sum tests. The second metric, *migration timing*, is based on the temporal progression of UID/day. Monthly binning provided a statistical framework within which significant changes and threshold crossings were identified. A threshold was defined at the 33^rd^ percentile of 1568 daily measurements of UID/day made across all years, effectively describing separation of the lower third from the upper two thirds of the statistical distribution of values. To consider the timing of northward spring migration, returning from distant breeding habitat to local foraging habitat, we identified the first month in which monthly median UID/day rose and remained above the threshold. To consider the timing of southward winter migration, away from local foraging habitat to distant breeding habitat, we identified the first month in which monthly median UID/day fell and remained below the threshold. The third metric, *site fidelity*, was based on the number of days within each year that each individual was identified in the study area. This individual-based statistic was examined at the interannual time scale via nonparametric boxplots and evaluation of significant differences using Wilcoxon rank sum tests. Sensitivity of statistical comparison was tested by screening individuals included in the analysis at a range of minimum thresholds for the number of days that each individual was identified within each year being compared: from 1 to 4 days.

**Fig 5:**
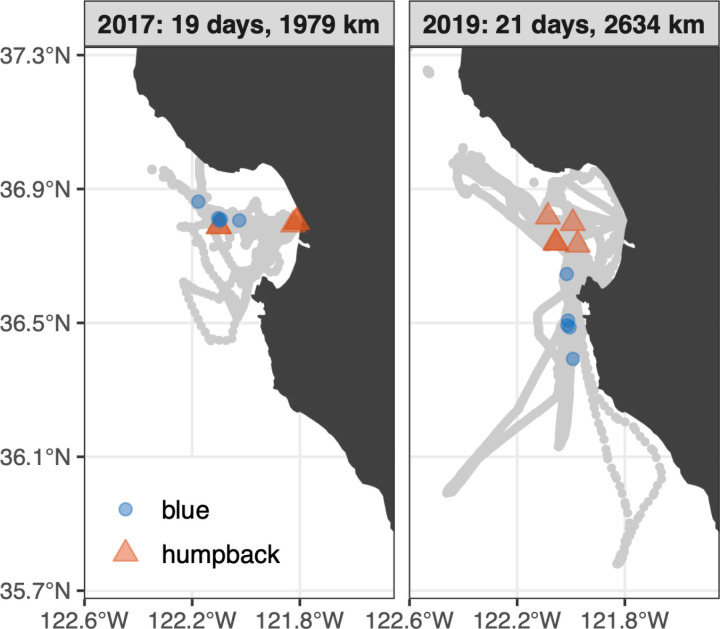
Scientific visual survey coverage. Survey tracks (gray) are represented for years that strongly contrasted in both humpback whale acoustic detection and forage species abundance and composition (see Results). Total survey distance and time for each year are summarized in the map annotations. Blue and red symbols show the locations of whale biopsy samples.

To complement the analysis of all available photo-ID data (Fig 4), we also examined sightings and photo-ID data acquired during regional scientific surveys [74]. This analysis examined fall 2017 versus fall 2019 within study years 3 and 5, which were marked by strong contrasts in acoustic detection of humpback whales and in the abundance and composition of forage species populations. Scientific visual surveys were conducted between August and November in 2017 during 19 days of effort along 1979 km, and between July and December in 2019 during 21 days of effort along 2634 km (Fig 5). Survey temporal coverage spans the annual period when humpback whale acoustic detection rises to its annual peak in the Monterey Bay region [29].

#### Regional and local forage species abundance and aggregation.

Standardized abundance data for forage species in the central CCE, based on annual midwater trawl surveys conducted between late April and mid June, were acquired through the Rockfish Recruitment and Ecosystem Assessment Survey (RREAS). For over 40 years the RREAS has sampled fixed stations and conducted trawls at night (when forage species are relatively shallow) with a target depth range of 30–40 m. These data have been applied to study variation in forage species in relation to ecosystem conditions and associated consequences for foraging ecology and ecosystem-based management [16,25,26,29,75–77]. The RREAS forage species categories that we focus on are krill (total species abundance), adult anchovies and adult sardines. Regional indices are derived from model-based estimates that account for sampling effort and distribution [78] and are provided to the California Current Integrated Ecosystem Assessment [79]. Observations of forage species upon which whales were feeding were also part of the scientific surveys described for humpback whale photo ID; these data are examined in relation to focal individuals for whom biopsy samples were collected (Fig 5; see section on dietary inference below).

Complementary to the strength of the annual spring-summer characterization of regional forage species by RREAS, moored echosounder data can provide continuous characterization locally to more fully assess the quality of the forage preyscape utilized by whales. During the last two years of the study period, observations of forage species were made nearly continuously with a 38 kHz upward-looking scientific echosounder at MARS, collocated with the hydrophone (Fig 1). This instrument could detect macrozooplankton, micronekton, and larger animals from its seafloor position at 890 m depth nearly to the sea surface. However, this echosounder is more sensitive to fish than krill and is therefore most appropriate to considering the foraging ecology of humpback whales. The modified Simrad EK60 echosounder transmitted a 2.048 ms ping upward in a 7-degree beam every 2.5 s using an output power of 400 W [80]. Data were processed using Echoview software to remove ambient noise and spikes along with other invalid data including the ocean surface before additional analyses that used a combination of Echoview and custom scripts in LabVIEW. Aggregations, contiguous areas of scattering that were significantly higher in intensity than their surroundings, were detected following [81]. Humpback whales in the CCE typically forage within the upper 200 m [82], therefore the total area scattering (s_A_, m^2^/nmi^2^) and the area scattering within aggregations in the upper 200 m were calculated for each day.

#### Dietary inference.

To consider changes in whale diet that may have resulted from interannual changes in forage species abundance and composition, we conducted a limited examination of stable carbon (*δ*^13^C) and nitrogen (*δ*^15^N) isotope values from biopsy samples. In this exploratory analysis we focused on humpback and blue whales because more biopsy samples exist for these species, and they are the most divergent in foraging behavior – with stenophagous blue whales contrasting the foraging flexibility of humpback whales. Our sample data set comprised five skin samples from each whale species for each of two years that strongly contrasted in forage species abundance and composition: 2017 versus 2019. Biopsy samples, including skin and a thin layer of blubber, were collected between July and September in the Monterey Bay region (Fig 5) by Cascadia Research Collective from small boats using a modified crossbow fitted with a hollow-tipped dart [83]. Samples were frozen at −80°C and stored at the Southwest Fisheries Science Center until processing. Prior to stable isotope analysis (SIA), 5 mg of skin from each sample was split, lyophilized (in a Virtis benchtop lyophilizer) and lipid extracted using a Dionex Accelerated Solvent Extractor (ASE using 100% petroleum at three 5-min cycles at 1500 psi and 100 °C) and homogenized before weighing out approximately 1 mg into a tin capsule for SIA. Sample analysis for *δ*^13^C and *δ*^15^N was conducted at the University of Florida Gainesville Stable Isotope Laboratory and used a Thermo Electron DeltaV Advantage isotope ratio mass spectrometer coupled with a ConFlo II interface linked to a Carlo Erba NA 1500 CNHS Elemental Analyzer. All carbon isotopic results are expressed in standard delta notation relative to VPDB. All nitrogen isotopic results are expressed in standard delta notation relative to AIR. Resulting isotopic signatures were compared using the Stable Isotope Bayesian Ellipses in R (SIBER) package [84] to examine differences between years and species. Isotopic niche widths of blue and humpback whales were calculated based on the standardized ellipse of the *δ*^13^C and *δ*^15^N data and an ellipse corrected for small sample size (SEA_c_).

***Ethics statement*:** Whale biopsy sampling and scientific surveys by Cascadia Research were conducted under NOAA National Marine Fisheries Service scientific research permit 21678 issued to JC.

#### Upwelling intensity.

To consider how upwelling drives interannual variation in nutrient supply and primary productivity, we examined the biologically effective upwelling transport index (BEUTI; [17]) computed for 37°N, immediately north of Monterey Bay (Fig 1). After smoothing the daily time-series with a 5-day running mean, cumulative upwelling for each year was computed as the sum of positive daily values. Persistent periods of downwelling (negative BEUTI) are associated with winter storms, during the annual period when primary productivity is light limited. The sum of positive BEUTI values thus effectively focuses on conditions following the annual spring transition to upwelling, when primary production rises with increasing nutrient supply and insolation [12], thereby fueling ecosystem productivity. A similar approach to characterization of annual BEUTI has been applied to examining interannual variations in the timing of blue whale migration in relation to upwelling phenology [19].

Coastal upwelling also affects baleen whale foraging ecology more directly through relationships to upwelling intensity. On the time scale of individual oscillations in upwelling intensity (days to weeks), blue whales in the Monterey Bay region have been observed to track the cool upwelling plumes within which krill aggregate [48,85]. We are not aware of studies that examine this association in fin whale populations. Over longer time scales, humpback whale habitat occupancy in the eastern North Pacific has been observed to respond strongly to marine heatwave conditions that compress cool upwelling-influenced habitat close to the coast, in turn exacerbating entanglement of whales in nearshore fishing gear [76,86]. We examine the areal extent of upwelling habitat to address a specific question: Was compression of cool (upwelling) habitat evident, and thus potentially influential, across two focal study years in which visual and acoustic detection metrics for humpback whales diverged significantly? Using sea surface temperature data from the monthly Multi-scale Ultra-high Resolution (MUR) SST Analysis fv04.1, acquired via the NOAA ERDDAP server, we compare the area off central California having SST below a threshold indicative of upwelling during spring (12.5°C). This isotherm was previously defined for computing the Habitat Compression Index (HCI) across seasons and regions of the CCE [86].

#### Environmental factors in relation to acoustic detection.

To examine how environmental factors may have influenced acoustic detection of whale song, we considered both acoustic masking and acoustic propagation. Noise levels in the background frequency bands of blue and fin whale call indices (Fig 3) were examined with graphical and statistical summaries (box plots and Wilcoxon rank sum tests) of daily mean power spectral density at seasonal and interannual time scales. Because humpback whale song extends across a frequency band impacted by wind noise [87,88], we also examined local wind speed data graphically and statistically. This analysis was constrained to the focal interannual comparison (Year 3 versus Year 5) for which the most comprehensive data (acoustic and visual detection metrics, stable isotope measurements from whale biopsy samples, environmental and ecosystem conditions) were examined to assess hypotheses.

For the focal interannual comparison of humpback whale song detection (Year 3 versus Year 5), we also modeled how interannual changes in oceanographic conditions may have influenced acoustic detection through changes in sound speed profiles and associated changes in acoustic transmission loss. After identifying that the interannual difference was driven by song detection during spring (March – May), we modeled acoustic transmission loss of humpback whale song calls at daily resolution during these months of both years. The acoustic model has been previously described and applied to characterize the spatial domain over which humpback whale song is received at MARS during the season of peak song occurrence [29], and the reader is referred to this prior study for model details and specification of sound source attributes. In the present study, we first confirmed the ability of the HYCOM model, upon which the acoustic propagation model relies, to represent observed interannual differences in environmental conditions. This involved comparing HYCOM model SST with MUR SST satellite remote sensing data. After confirming HYCOM’s accurate representation of interannual changes, we modeled acoustic transmission loss profiles daily at 100 m horizontal resolution along a 110 km section extending both inshore and offshore of the MARS observatory. The distributions of acoustic transmission loss between MARS and a humpback whale singing at 20 m depth (consistent with observations [87]) were examined graphically and statistically tested for differences (boxplot and Wilcoxon rank sum tests).

## Results

### The measured acoustic signals represent song

Interpretation of measured acoustic signals as song is supported by different analyses for each species. Validation of daily presence/ absence of humpback whale song from CNN results, based on examination of two years of high-resolution spectrograms (November 2018 through October 2020), yielded performance metrics of 100% precision and 97% recall. Blue whale A call occurrence was confirmed for 96% of all days for which the daily B-call metric defined song presence, and A and B call cooccurrence within song sessions was consistently apparent in the high-resolution spectrograms. For some of the remaining 4% of days, faintness of the song signal and presence of background noise, in addition to the relatively weak energy of A calls compared to B calls (Fig 2), may underlie the inability to confirm A call cooccurrence. Examination of high-resolution spectrograms also showed that from the time fin whale song is first detected in fall through the time it is last detected in spring, these low-frequency pulses consistently occur in series having a regular interpulse interval that defines song [71]. These analyses thus support the interpretation of quantified acoustic signals from all three whale species as song.

### Seasonal patterns

**Fig 6:**
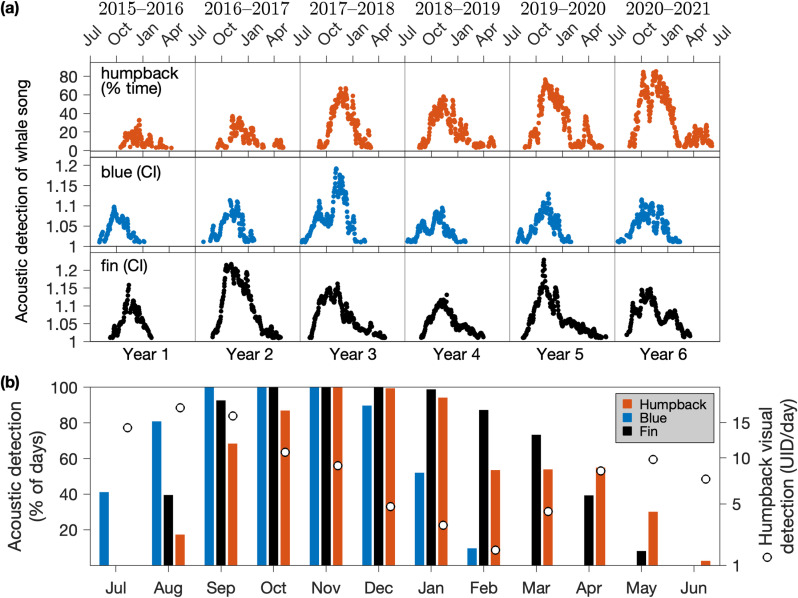
Seasonal variations. (a) Daily values of song detection metrics are the percent of recording time during which a deep convolutional neural network [67] detected humpback whale song and the call index (CI) for blue and fin whales (Fig 3). Points shown are above the minimum threshold of the full time-series: more than 3% of daily recording time with a model score exceeding 0.7 for humpback whales, or CI > 1.01 for blue and fin whales. (b) A climatology of seasonal variation in song detection derived from the data in (a), pooled by month across years. Bars represent the percent of recorded days within each month during which song detection exceeded minimum thresholds. For humpback whales, circles (right axis) represent the monthly mean number of unique photo IDs per day, derived from data spanning the full study period (Fig 4).

Monthly statistics derived from all years of data (Fig 6) show differences in song phenology among species, with blue whale song offset earlier (July - February) and detectable for a shorter period (8 months) compared to fin whale song (August - May, 10 months) and humpback whale song (August - June, 11 months). Also examined at climatological monthly resolution, photo ID data for humpback whales (Fig 4) allow comparison of seasonal visual and acoustic detection metrics (Fig 6b). While mean rates of humpback visual detection are highest during July through September, song detection rises steeply from 0% to 68% of days, indicating emergence of this acoustic behavior within the regional foraging population. Visual detection declines steadily during October through February, with the mean dropping from 11 to 2 UID/day as part of the population migrates away from local foraging habitat to distant breeding habitat. Concurrently, song detection rises to an annual peak at 100% of recording days during November before declining to 54% of recording days by February. Visual detection rates increase from the annual minimum of 2 UID/day in February to 10 UID/day in May as whales return to foraging habitat, while song detection rates remain at intermediate levels: 54% in March, 55% in April, and 30% in May. Disappearance of song detection during June–July occurs amid stable and high levels of visual detection, indicating cessation of this acoustic behavior.

### Interannual patterns

**Fig 7:**
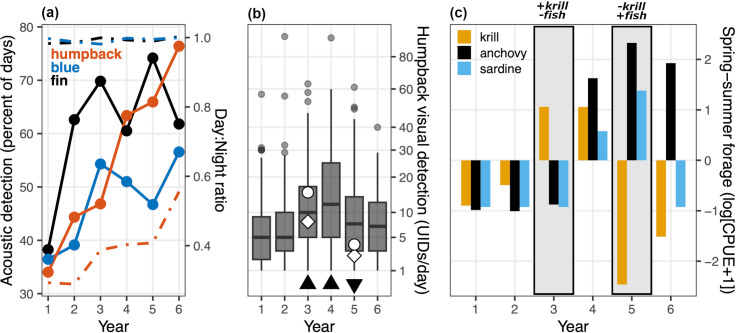
Interannual variations. (a) Annual metrics of acoustic detection (solid lines, left axis) and its day:night ratio (dashed lines, right axis) for each whale species. (b) Boxplot of the daily number of unique humpback whale photo IDs from data in the Monterey Bay region (Fig 4), pooled by year (beginning July 1, as in Fig 6a). Shown are the interquartile range (IQR, box), median (thick horizontal line), minimum (lower limit of vertical line), the third quartile + 1.5 × IQR (upper limit of vertical line), and outliers (gray circles). Solid black triangles along the bottom indicate significant increase (years 3,4) or decrease (year 5) in daily unique humpback whale IDs relative to the previous year (p < 0.05; one-sided Wilcoxon rank sum tests). Scientific visual survey data in the Monterey Bay region during summer-fall of years 3 and 5 (Fig 5) show the number of unique individuals encountered per day (white diamonds) and per 50 km of survey track (white circles). (c) Forage species abundances off central California [79] represent spring-summer forage conditions leading into the start of each annual song period (Fig 6a). Years 3 and 5 are highlighted because of their strongly contrasting relative abundances of krill versus forage fish, which are considered with regard to acoustic and visual detection of humpback whales (Figs 7a, 7b, 8) and dietary inference from stable isotope analysis of biopsy samples (Fig 9).

Interannual variation in song detection was pronounced (Fig 7a). During years 1–3, the annual metrics show that all three whale species exhibited a positive trend in acoustic detection, increasing by factors of 1.4 (humpback),1.5 (blue) and 1.8 (fin). Blue and fin whales exhibited correspondence between the magnitude of interannual increase and the nature of the underlying daily metrics. The years showing the highest daily CI for the longest duration (Fig 6a, year 2 for fin whales; year 3 for blue whales) were the same years that exhibited the greatest increase in the percent of days having acoustic detection (Fig 7a). During years 4–6, humpback whale acoustic detection uniquely continued to increase each year, reaching levels higher by a factor of 2.2 during year 6 than year 1. This distinction of humpback whale song was uniquely paralleled by a positive trend in the day:night ratio of song activity, increasing by a factor of 1.9 during the study period (Fig 7a). Neither blue nor fin whales exhibited a continuous positive trend in song detection, instead showing decreases across two of three interannual changes following year 3. They also exhibited no significant changes in annual day:night ratios of song activity (Fig 7a).

Humpback whale photo ID data allowed consideration of local population abundance, site fidelity and migration timing as potential factors in acoustic detection. The metric for local population abundance indicated significant interannual increases in two sequential year-to-year comparisons: years 2–3 and 3–4 (Fig 7b), consistent with increasing acoustic detection (Fig 7a). This visual metric subsequently decreased between years 4 and 5 and did not change between years 5 and 6 (Fig 7b), inconsistent with continuing increases in acoustic detection of song and its daytime proportion (Fig 7a). The metric for site fidelity showed a significant increase in one year-to-year comparison, between years 2 and 3 (*p* < 0.01). In all other comparisons, *p*-values for Wilcoxon rank sum tests exceeded 0.45 regardless of the screening of individuals included in the analysis (based on a minimum number of days of identification within each year).

The metric for migration timing indicated that interannual variation in the months during which humpback whales maintain substantial levels of presence within Monterey Bay depends more upon the timing of the northward migration into local foraging habitat in spring than the timing of the southward migration out of local foraging habitat in winter. A key reference point is the month during which median UID/day reached an annual minimum, presumably representing when the largest proportion of the regional population was away from foraging habitat due to the breeding migration; this month was February in all years. Considering the winter migration period, monthly median values of the daily metric fell and remained below the threshold of 4 UID/day in December of all years, except year 2 when this transition occurred one month later. Considering the spring migration period, monthly median values of the daily metric rose and remained above the threshold of 4 UID/day beginning within the 3-month period of March through May in the following sequence for years 1–6: April, May, March, March, April, May. This interannual variation exhibited no trend toward earlier return to foraging habitat in a way that could have determined the positive trend in acoustic detection. Taken together, these metrics of migration timing can quantify the number of months within each year that median UID/day was above the 33^rd^ percentile threshold: 8, 8, 11, 9, 9, and 7 months, respectively. Increased duration of UID/day above the threshold is indicated for only one year-to-year comparison: years 2–3. Further, the year with the fewest months of sightings above the threshold, year 6, had the highest level of song detection (Fig 7a).

### Foraging ecology

Large interannual variations in forage species abundance and composition are indicated by annual spring-summer sampling off central California during the study period. During the first three years, abundance indices of forage fish (anchovy and sardine) were persistently low while krill stocks steadily increased from negative, to near-neutral, to positive anomalies (Fig 7c). This trend in krill abundance paralleled increases in biologically effective upwelling (Fig 1c) and primary productivity during regional emergence from a multi-year marine heatwave [29]. All three whale species consume krill, and song detection for all three whale species increased during these years. During the latter three years of the study period, krill abundance transitioned to strongly negative anomalies while at least one forage fish species exhibited strong positive anomalies (Fig 7c). Of these two fish species, anchovy populations were much more abundant in absolute terms, and positive anomalies in their abundance persisted throughout all three years. During this period humpback whales uniquely showed a continuing positive trend in both song detection and the proportion of daytime song (Fig 7a). In contrast, blue and fin whales showed decreases in song detection in two of three interannual comparisons.

**Fig 8:**
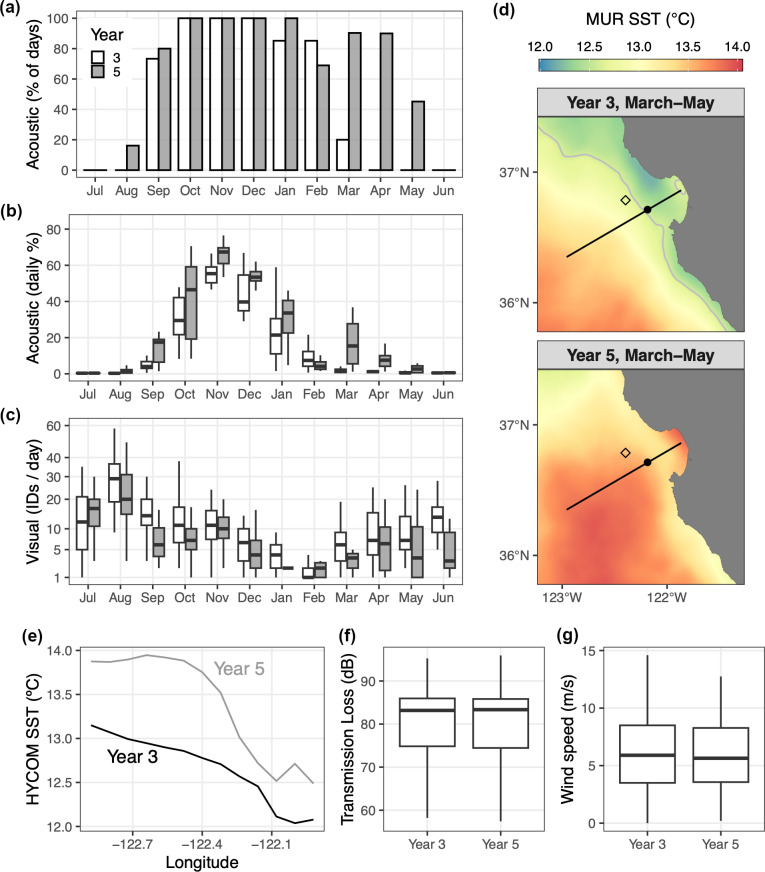
Humpback whales: seen less but heard more. Comparison of acoustic and visual metrics of humpback whale detection during years of contrasting forage species composition and abundance (Fig 7c), years 3 and 5. The acoustic summaries (a, b) were derived from the data shown in Fig 6a; the visual summary (c) was derived from the data shown in Fig 4. (d) Average regional sea surface temperature (SST) for March through May of years 3 and 5, from satellite remote sensing; the gray contour is the 12.5°C isotherm. The black transect line represents the vertical section along which acoustic transmission loss was modeled. (e) March–May average HYCOM SST along the transect shown in (d). (f) Boxplot of acoustic transmission loss between a humpback whale calling at 20 m depth along the transect shown in (d) and the MARS observatory. (g) Boxplot of hourly wind speeds measured at NDBC Station 46042 (location shown by the diamond in (d)). Boxplots (b,c,f,g) show the interquartile range (IQR, box), median (thick horizontal line), minimum (lower limit of vertical line), and third quartile + 1.5 × IQR (upper limit of vertical line).

Years 3 and 5 show particularly strong contrasts that are considered in greater detail for humpback whales, the species for which we have the most substantial data resources. Year 5 showed significantly lower visual detection overall (Fig 7b, *p* < 0.01), two fewer months with UID/day above the threshold defined for migration timing, and no photo-ID evidence of increased site fidelity (*p* < 0.01). The decrease in visual detection is indicated by not only persistent observations concentrated within Monterey Bay – owing to the spatial footprint of ecotourism (Fig 4b, boxplot in Fig 7b), but also by scientific surveys in the broader Monterey Bay region (Fig 5, open symbols in Fig 7b). Despite these consistent visual metrics, acoustic detection increased significantly (*p* < 0.01) by a factor of 1.4, from 47% of days in year 3 to 66% of days in year 5 (Fig 7a). This pattern of humpback whales being seen less but heard more in year 5 versus year 3 was evident in multiple ways (Fig 8). During year 5, song was detected more during both fall (August) and spring (March–May) and with greater daily levels of intensity in most months (Fig 8a, 8b), in strong contrast to persistently lower levels of visual detection (Fig 8c). The greatest divergence between detection methods occurred during March–May, when acoustic detection increased from 6% of days in year 3 to 75% of days in year 5 (Fig 8a) while visual detection decreased significantly (Fig 8c, *p* < 0.01). SST < 12.5 °C was not observed within the study region during March–May of year 5, a condition indicative of habitat compression [76,86]. This would tend to shift humpback whale foraging habitat toward the coast, which in turn would presumably elevate sightings within the bay where our photo-ID data are concentrated (Fig 4). However we observed the opposite, lower levels of sighting in the bay during the habitat compression of year 5. Thus the potential role of habitat compression on the divergence of visual and acoustic detection metrics is not supported.

The HYCOM model accurately represented interannual changes in springtime SST distributions (Fig 8d, 8e), however modeling of acoustic transmission loss showed no significant interannual difference (Fig 8f). Observed wind speeds during these two spring periods also showed no significant difference (Fig 8g). The inability to explain increased acoustic detection during year 5 based on physical factors (propagation, masking) or independent visual metrics (local abundance, site fidelity or migration timing) motivates consideration of changes in the acoustic behavior of humpback whales, which may follow from large changes in foraging ecology (Fig 7c).

**Fig 9:**
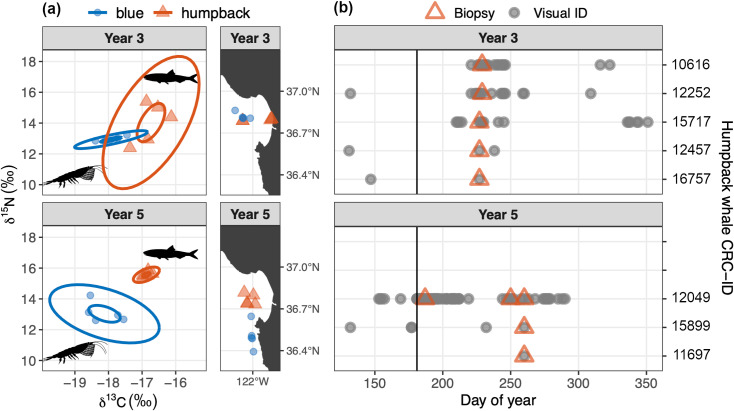
Dietary inference from whale biopsy samples. (a) 40% and 95% ellipses of the *δ*^13^C and *δ*^15^N data represent isotopic niches of blue and humpback whales sampled in year 3 (fall 2017) and year 5 (fall 2019). Adjacent maps show the locations of sampled whales, five samples from each species in each year; darker symbols indicate overlapping location markers. (b) Local sighting histories of humpback whales sampled for biopsy during study years 3 and 5 (Table 2).

Isotopic signatures measured from whale biopsy samples inform understanding of their foraging ecology. Overall, observed differences between blue and humpback whales are consistent with species-dependent differences in diet. Blue whale isotopic signatures exhibited lower *δ*^15^N and *δ*^13^C compared to humpback whales (Fig 9a, Table 1), reflective of the strict krill-based diet of blue whales compared to the krill and fish diet of humpback whales. Interannual changes in isotopic signatures followed variations in forage species. Widening of the blue whale isotopic niche between years 3 and 5 (Fig 9a) suggests foraging over a relatively larger region when krill abundances were low in year 5 (Fig 7c), as *δ*^13^C varies across geographic gradients [89,90]. Slight overlap between isotopic niches of blue and humpback whales during year 3 (Fig 9a, 95% ellipses show 3% overlap of the total isotopic space) mirrored direct spatiotemporal overlap of sampled animals. Proximity of humpback and blue whales sampled in the outer bay during year 3 was close both spatially (Fig 9a) and temporally (humpback whales were sampled on 15 August; blue whales were sampled during 13–16 August). The wider isotopic niche of humpback whales in year 3 versus year 5 (Fig 9a) is consistent with foraging on both krill and fish when + krill | –fish anomalies were observed (Fig 7c). Visual observations in year 3 showed that humpback whales were foraging on fish where they were sampled within inner Monterey Bay, and on krill where they were sampled in the outer bay near blue whales (Fig 9a, Table 2). The much narrower isotopic niche indicated for humpback whales in year 5 (Fig 9a) is consistent with a fish dominated diet when –krill | + fish anomalies were observed (Fig 7c). Of the year-5 biopsy samples, three were from the same individual – *Fran* (Fig 4a). Sampled across 74 days in year 5, *Fran* (CRD-ID 12049 in Fig 9b) exhibited a stable isotopic composition indicating a fish-dominated diet (Fig 9a).

**Table 1:** Results of stable isotope analysis of blue and humpback whale biopsy samples.

	*δ*^15^N ‰	*δ*^13^C ‰	TA	SEA	SEA*c*
**Year 3 (2017)**					
**blue**	12.93	−17.93	0.11	0.11	0.15
**humpback**	14.03	−16.74	1.58	1.53	2.04
**Year 5 (2019)**					
**blue**	13.12	−18.16	0.81	0.85	1.14
**humpback**	15.59	−16.86	0.06	0.05	0.07

*δ*^15^N and *δ*^13^C are mean values from all samples in each category. TA: total isotopic niche area, SEA: standard ellipse area, SEAc: standard ellipse area corrected for small sample size [84].

**Table 2:** Individual data on humpback whales examined for dietary inference.

CRC-ID	Body length (m)	Date(s) sampled	Location	In situ prey observations	Σ Days identified locally
**Year 3 (2017)**					
**10616**	13.13	17-Aug-2017	nearshore	anchovy	17
**12252**	11.69	17-Aug-2017	nearshore	anchovy	16
**15717**	–	15-Aug-2017	nearshore	–	16
**12457**	12.21	15-Aug-2017	offshore	krill, lunge feeding	3
**16757**	11.19	15-Aug-2017	offshore	krill	2
**Year 5 (2019)**					
**12049**	–	3 dates: 6-Jul, 7-Sep, 17-Sep-2019	offshore	–	64
**15899**	–	17-Sep-2019	offshore	Feeding with California Sea Lions, likely fish	5
**11697**	–	17-Sep-2019	offshore	–	1

Dashes indicate that no data were available.

Continuous echosounder data from the final two years of this study reveal changes in prey that are particularly relevant to humpback whales. This echosounder is more sensitive to fish than krill, and the dominant forage fish, anchovy, were abundant during both years (Fig 7c). The echosounder data show increased daily total scattering in the upper 200 m (1.7× median difference), indicating increased total prey abundance. They also show increased total scattering in aggregations (4.4× median difference), representing increased occurrence of dense prey patches. Aggregative swarming of krill and forage fish in response to strengthening of coastal upwelling has been documented in this region [85]. Swarming that increases prey density within patches can, in turn, increase the efficiency of trophic transfer between prey and the predators that consume them. The intensification of upwelling between years 5 and 6 (Fig 1c) thus indicates that changes in forage species behavior (aggregation), not just abundance and composition, may have been influential across these years in which acoustic detection of humpback whales increased significantly (Fig 7a, *p* < 0.01) without a change in visual detection (Fig 7b, *p* < 0.01).

## Discussion

Considering the vast oceanic areas inhabited by mysticetes and the efficient transmission of sound in the sea, passive acoustic monitoring is naturally an effective tool for detecting Earth’s largest soniferous animals. Baleen whale song is a prevalent acoustic behavior off central California, detectable during any month of the year for at least one of the three species examined in this study – blue, fin, and humpback. The prevalence of song makes this particular acoustic behavior an effective focus of biological remote sensing. However, understanding the complex ecology that underlies the presence and behavior of whales is challenging and requires integration of diverse methods of sensing the animals and their dynamic ecosystem. The Monterey Bay region is an important foraging habitat for mysticetes that inhabit the eastern North Pacific [10,91], and previous studies in the Monterey Bay region have shown how passive acoustic monitoring can reveal underlying influences of foraging ecology on the acoustic behavior and acoustic detection of baleen whale species. These studies have examined the role of krill aggregation dynamics in the foraging strategies and movement ecology of blue whales [48,92,93], the consequences of large interannual changes in productivity and foraging ecology for the timing of blue whale migration [19,49], and the behavioral ecology of humpback whales amid extreme ecosystem variations [29]. In the present study we applied interdisciplinary observations to expand, temporally and taxonomically, examination of the behavioral ecology of three sympatric mysticete species with overlapping niches in regional foraging ecology.

### Seasonal patterns

Song detection in all three whale species begins annually during the late summer/ early fall (Fig 6). The rise of humpback whale song detection beginning in August follows high levels of visual detection during April–July, indicating that the annual rise of song represents emergence of this acoustic behavior from an actively foraging population (Fig 6b). Blue whales are also present in the CCE during summer, acoustically detected by their foraging-associated D calls typically by May in the Southern California Bight [34] and the Monterey Bay region [68]. The rise of song in blue whales during July thus similarly suggests a change in acoustic behavior, specifically males beginning to sing. However, visual data indicate primary months of blue whale sighting in the Monterey Bay region during July through October [32,91], and the rise of blue whale song in July may be influenced by both increased local population abundance and seasonal change in acoustic behavior. Fin whale sighting data off central California is limited, however a five-year study within a large region off southern California showed that fin whales were visually detected frequently beginning in July, similar to blue whales [94]. In this context, the emergence of fin whale song during August in the Southern California Bight [37] and central CCE (this study) may similarly be driven by change in acoustic behavior and not simply increased local abundance.

Consistent with a life history strategy of capital breeding, the annual winter decline of song detection in this foraging habitat reflects migration to distant low-latitude breeding habitat [19,33,38,39,49]. While blue and fin whales show a single decline in acoustic detection of song each year, humpback whales uniquely exhibit a bimodal pattern with secondary peaks in song detection during spring (March–May) that are lower than the fall peak and variable from year to year with regard to occurrence, timing, duration, and intensity (Fig 6a). Humpback visual sightings consistently reached their annual minimum in February of every year in the study period, and we speculate that these variable secondary peaks in song detection during March–May represent whales returning to foraging habitat in the spring, continuing their singing behavior that peaks in the breeding season. This interpretation is consistent with continual singing of humpback whales into late spring that has been documented for foraging habitat in the northwest Atlantic [95] as well as singing during migration from breeding to foraging habitat in the eastern North Pacific [61].

The longer annual duration of song detection from fin (10 months) and humpback (11 months) whales (Fig 6b) compared to blue whales (8 months) is consistent with visual sighting data off southern California, which showed absence of blue whales in winter and spring, in contrast to humpback sightings in spring through fall and year-round sightings of fin whales [94]. More persistent acoustic detection of fin whales compared to blue whales has been observed in the northeast Pacific [96] and associated with year-round presence in the Southern California Bight [36,37]. Regardless of these differences, all three species demonstrate prevalent and persistent singing in foraging habitat, and this prevalence enables accurate detection of song to be an effective data resource for ecological research in foraging habitat.

### Interannual patterns

Interannual variability in song detection during the study period was large, varying by factors of 2.2 (humpback), 1.6 (blue) and 1.9 (fin). The primary difference across species was multifaceted distinction of humpback whales, which showed the largest range in song detection, the only continuously positive trend in song detection, and the only positive trend in the proportion of song occurring during the day. The primary similarity across species was increased song detection during the first three years, rising out of the first-year minimum. Acoustic recording began near the peak of a multi-year marine heatwave [20,97], which was associated with low levels of primary productivity and major food web consequences [21,24,29]. Beyond changes is trophic energy transfer, high levels of food web toxicity during an extreme harmful algal bloom (HAB) in the northeastern Pacific during 2015 caused the most widespread poisoning of marine mammals ever documented, including whales [22]. As observed in our study region, this HAB and its underlying physical, chemical and biological anomalies were extreme [23].

Initiation of this time series during the height of extremely anomalous ecosystem conditions suggests that the common rise of song among all three whale species during years 1–3 represents recovery from exceptional conditions to more typical conditions. Key ecosystem factors considered in a prior study examining only humpback whale song were increases in primary productivity and krill abundance, in parallel with decreases in food web toxicity driven by phytoplankton ecology [29]. In this context, one discovery of the present study – that acoustic detection of blue and fin whales increased in parallel with that of humpback whales during three years of emergence from a heatwave – is consistent with ecosystem-wide impacts of this major environmental perturbation. Marine heatwave influence on the acoustic behavior of blue whales has been inferred from PAM and environmental observations in the South Taranaki Bight region of Aotearoa New Zealand, where whales exhibited reduced foraging and reproductive effort during a heatwave [30].

Possible factors influencing interannual variation in acoustic detection at a fixed location are diverse and may be interrelated. These factors include local population abundance, local site fidelity, duration of annual residence in foraging habitat (migration timing), proximity of calling whales to the acoustic receiver, demographic composition of the local population (proportion that are mature males), acoustic behavior of mature male whales, and the extent of attenuation or masking by environmental conditions. Data resources, and hence the ability to examine hypotheses about causal factors, differ among the whale species in this study. We consider each of these species in turn, starting with the species for which we have the greatest data resources – humpback whales.

#### Humpback whales.

The relatively greater potential to consider factors driving interannual variations in humpback whale song detection is largely due to extensive photo ID data that were uniquely available for this species from the full study period. These data leverage extensive sampling from ecotourism and research activities and were acquired from within MARS’ acoustic detection range [29]. The data do not comprehensively define age and sex of all identified whales, as would be required to estimate abundance of the local adult male population that presumably would be the primary source of song signal [43]. However, knowledge of individual identity from photo ID enables quantification of the number of unique individuals and how this metric changes through time. Compared to simply the number of sightings of a species, the ID-based metric more accurately represents local animal abundance and is more directly comparable to acoustic detection. This photo ID data informed examination of three potential factors for all sequential year-to-year changes in song detection: local population abundance, site fidelity, and migration timing (Table 3).

**Table 3:** Assessment of factors having potential influence on interannual changes in humpback whale song detection.

Factor	Yrs 1–2	Yrs 2–3	Yrs 3–4	Yrs 4–5	Yrs 5–6	Total ^+^
**Local site fidelity**		+				1
**Migration timing**		+				1
**Local population abundance**		+	+			2
**Foraging ecology/ efficiency**	+	+	+	+	+	5

+ symbols indicate that this study found support for the conclusion that a given factor may have contributed to increased acoustic detection in one year relative to the previous year.

The potential influences of local site fidelity or migration timing on the continuous positive trend in humpback whale song detection are indicated to be minimal. There was only one interannual increase in the metrics representing these factors, years 2–3 (Table 3). The potential influence of local population abundance on the continuous positive trend in humpback whale song (Fig 7a) is supported to a moderate extent, for two of the five sequential interannual comparisons: years 2–3 and 3–4 (Table 3, Fig 7b). However, comparison of visual and acoustic detection metrics also illustrates how they can diverge significantly. The large increase in acoustic detection across the focal comparison of year 3 versus year 5 coincided with decreases in local visual detection metrics derived from both the greater aggregated photo-ID dataset and regional scientific surveys (Fig 7a, 7b). Throughout the annual cycle of song occurrence in these years, humpback whales were persistently heard more but seen less during year 5 (Fig 8a–8c), and the period when these metrics were most divergent across years was during the spring (March–May). Despite habitat compression that was evident during year 5 (Fig 8d), which would cause humpback whales to forage closer to the coast [76,86] and could have elevated sighting levels in the bay (Fig 4), we observed the opposite – lower sighting levels during year 5 (Figs 7b, 8c). Therefore, comparison of acoustic and visual detection metrics (Fig 8a–8c) was unlikely to have been biased by environmental forcing of habitat compression. Instead, the degree to which humpback whales were seen less but heard more during year 5 may be underestimated in this comparison. Also for this focal comparison of years 3 and 5, we found no indication of influence from changes in acoustic propagation or masking by wind noise (Fig 8f, 8g). This integrative examination thus points to considering changes in acoustic behavior. Foraging is the most essential life activity in this habitat, and changes in foraging ecology have diverse consequences for whale behavior.

Changes in foraging efficiency, driven by major changes in forage species populations, may influence prey search effort, habitat occupancy, health, energy storage on short to cumulative long-term time scales, and allocation of time to different behaviors. Changes in acoustic behavior can be indicated by divergence of acoustic and visual detection metrics, and such divergence was evident in three of the five sequential year-to-year changes: years 1–2, 4–5, and 5–6 (Fig 7a, 7b). Increases in acoustic detection relative to visual detection may indicate increased time allocation to singing behavior. Variability in forage species abundance and composition in the central CCE was consistent with increasing foraging efficiency and potential consequences for acoustic behavior across all sequential year-to-year comparisons (Table 3). Increases in krill abundance during the first three years may have increased foraging efficiency amid persistent negative anomalies in forage fish abundance (Fig 7c). Increases in forage fish abundance and aggregation during the latter three years may have increased foraging efficiency for humpback whales particularly, because they are known to readily switch between available prey [26], and a fish-dominated diet would presumably be more energy rich. Measured wet caloric densities of prey sampled in the study region were 2–3 times greater for anchovies and sardines compared to krill [98]. Measurements of forage species sampled in the California Current Ecosystem have shown that Northern anchovy (*Engraulis mordax*) is the most energy dense and nearly twice that of the dominant krill species, *Euphausia pacifica* and *Thysanoessa spinifera* [99].

A shift toward a more fish-dominated diet in local humpback whales is indicated by the isotopic compositions of biopsy samples across the strongly contrasting years of forage abundance and composition: year 3 versus year 5 (Fig 9). Further, an individual sampled three times across 74 days of the foraging season in year 5 exhibited isotopic compositions indicative of a strongly and persistently fish-dominated diet (Fig 9, CRC-ID 12049), suggestive of preference for fish when forage abundance anomalies were –krill | + fish. The single largest increase in humpback whale acoustic detection, by a factor of 1.35, occurred between years 3 and 4. Although increased local population abundance may partially explain this increase (Fig 7b, Table 3), this was the only interannual change involving coincident occurrence of positive anomalies in the abundance of krill and both forage fish species. Humpback whales would presumably benefit uniquely from this scenario, as they can readily consume all of these prey.

Unique data resources for the final two years of the study period allow further consideration of the potential influence of foraging efficiency on humpback whale behavior and acoustic detection. Active acoustic sensing showed that increases in the local abundance (1.7×) and aggregation (4.4×) of forage fish, both of which would presumably enhance foraging efficiency, coincided with large increases in humpback whale song detection and its daytime proportion (Fig 7a). This occurred despite no indications from visual detection of increased humpback whale local population abundance, site fidelity, or persistence in foraging habitat between migration periods (Table 3). A physical basis for greater abundance and aggregation of forage fish is evident in the regional upwelling index, which increased significantly between these years (Fig 1c). While enhanced primary productivity from greater upwelling nutrient supply may support a larger population of forage fish, behavioral response of forage fish to upwelling – forming dense aggregations [85] – may increase the efficiency of prey capture and energy gain by whales.

The uniquely increasing proportion of song detection during the day for humpback whales, nearly doubling over the six-year period, was driven by two large interannual changes: between years 2 and 3 and years 5 and 6 (Fig 7a). Unlike the primary annual acoustic metric that emphasizes prevalence of detection within a year, the day:night metric indicates changes in diel patterns of acoustic behavior. An increased proportion of song detection during the day could represent changes in time allocation to primary behaviors, with more efficient daily foraging effort allowing more daily time allocation to singing behavior. The depths at which humpback whales lunge feed in the study region, measured using animal-borne tags, show a dominant peak deeper than 100 m [82]. This is consistent with the diel vertical migration of krill and other prey species that reside at depth during the day to avoid visual predators and thus is consistent with extensive foraging effort by humpback whales at depth during the day. The apparent importance of daytime foraging makes an increasing proportion of song detection during the day meaningful with regard to humpback whale behavioral ecology and relationships between foraging and singing behaviors.

Enhancement of foraging efficiency and energy storage, particularly early in the foraging season before singing behavior begins, may cumulatively increase the potential for time and energy allocation to song after this behavior emerges. This hypothesis can be most carefully considered for the interannual comparison having the greatest data resources: years 5 and 6. Increases in forage fish abundance and aggregation between years 5 and 6, detected from nearly continuous active acoustic sensing, are consistent with this hypothesis. Proportionally greater detection of song during the day may follow proportionally lesser foraging effort during the day. Evidence of seasonally diminishing daytime foraging rates has been found using deployment of biologging tags on humpback whales along the Western Antarctic Peninsula [100]. This study showed a seasonal progression toward feeding less often, at lower rates, and at deeper depths. These changes were interpreted to result from intensive foraging required to meet energetic needs when first transitioning from fasting to foraging, and from seasonal changes in krill availability as prey depth increases seasonally, however the potential role of intrinsic factors like satiation were considered.

Over longer time scales, fish-rich years may also allow whales to accumulate greater energy stores that support their long-distance breeding migration. In this context, greater secondary spring peaks in song detection during fish-enriched years in the CCE could reflect relatively greater energy stores, such that adult male whales returning from breeding habitat had healthier body conditions and more energy available for a full behavioral spectrum. Viewed from the other end of the range of environmental conditions, extremely poor foraging conditions – as observed early in our study period – would demand much greater foraging effort. This, in turn, would likely reduce energy gain, energy storage, and energy and time dedicated to behaviors other than foraging. Multiple hypotheses were considered with regard to the causality of large decreases in detection of humpback whale song within breeding habitat around Hawaii during a period when a marine heatwave strongly affected their foraging habitat in the northeastern Pacific [51]. These include decreased population size, shifts in the distribution of the population, altered migration patterns, and behavioral changes in (singing) males. Our study began during the same marine heatwave, and our observations within foraging habitat of the northeastern Pacific substantially support the role of changes in the behavior of male humpback whales.

#### Blue whales.

Interannual variation in blue whale song spanned a smaller range than that of fin and humpback whales (Fig 7a), consistent with their shorter annual period of song detection (Fig 6b). Within the full study period the greatest increase in annual blue whale song detection occurred between years 2 and 3 (Fig 7a). This largest increase occurred in the year marked by the greatest and most persistent elevation of daily CI (Fig 6a, year 3). These patterns suggest relatively high local population abundance and site fidelity, and this interpretation is supported by observations of dense aggregations of foraging blue whales within the study area during year 3 [92,93]. Potential influence of the timing of blue whale migration is suggested by results of a prior study for the same period, which showed interannual differences of up to four months in the timing of blue whale southward migration [19]. During the first three years, blue whale migration from local foraging habitat to breeding habitat occurred later each year, consistent with our finding that blue whale acoustic detection in local foraging habitat trended upward during these years (Fig 7a). However, during the subsequent three years, southward migration by blue whales occurred later in the year than during all of the first three years [19], inconsistent with decreases in blue whale acoustic detection during two of the three years (Fig 7a). Migration timing is thus not consistently supported in explaining interannual variation in acoustic detection of blue whales.

The role of changes in foraging ecology, and its potential modulation of whale acoustic behavior, is indicated by regional forage species data and stable isotope data from blue whale biopsy samples. As for both other whale species, the rise in blue whale song detection during the first three years paralleled emergence from the marine heatwave and recovery of krill populations. Consistent with being obligate krill predators, unlike humpback whales, blue whales exhibited a somewhat closer coupling to the interannual variations in krill abundance. The initial peak in song occurrence coincided with the initial peak in krill abundance during year 3, and a minimum in song occurrence during the remainder of the time series coincided with the return of strongly negative anomalies in krill abundance during year 5 (Fig 7a, 7c). The comparison of years 3 and 5 shows the largest difference in krill abundances, from the highest level of the study period during year 3 to the lowest during year 5 (Fig 7c). The parallel decrease in blue whale song detection may simply reflect lower local whale population abundance during the krill-impoverished year. However, isotopic compositions of blue whale biopsy samples were consistent with interannual changes in foraging ecology that could affect the behavioral spectrum of blue whales. Specifically, the broader range of variation in *δ*^13^C during year 5 (Fig 9a) is consistent with foraging over a relatively larger geographic domain when krill abundances were low, as *δ*^13^C varies across geographic gradients [89,90], and it coincided with lower levels of local acoustic detection. If relatively unfavorable foraging conditions in year 5 required blue whales to forage over larger spatial scales, this could have reduced acoustic detection in at least two ways: (1) more time and energy allocated to prey search could have made less time and energy available for singing behavior, and/or (2) more ephemeral habitat occupancy could have reduced the total time that whales were within the range of acoustic detection from the fixed monitoring location.

#### Fin whales.

The dominant interannual pattern of fin whale song detection was a steep rise, from 38% to 70% of days, during the first three years. Subsequently, the level of detection varied by less than 10% around the level reached in year 3. This dominant acoustic signal during the first three years is consistent with the dominant environmental variation of the study period, emergence from a marine heatwave (Fig 1c). Emergence from the heatwave was accompanied by recovery of krill populations from strongly negative anomalies to strongly positive anomalies (Fig 7c). Increased regional abundances of krill, a primary prey resource of fin whales [35], could have influenced acoustic detection through any of the factors listed at the start of the Discussion section, however none of these factors can be examined from available data resources.

### Trophic niches and evolutionary considerations

Comparison of blue and humpback whale trophic niche characteristics across a major food web transition indicate unique adaptations of these predators to interannual variations, as well as consideration of evolutionary pressures shaping their behavioral ecology. In year 3 humpback whales had a broader trophic niche that partially overlapped with krill-specialist blue whales (Fig 9a). The humpback whales observed to be foraging on krill in year 3 were sampled in close proximity to blue whales that aggregated in exceptionally dense groups at a krill foraging hotspot [92,93]. In year 5 humpback whales had a narrow trophic niche that did not overlap with blue whales, despite the fact that the blue whale isotopic niche area expanded between these years (Fig 9a). These changes in trophic niche overlap emphasize the advantage of prey switching [26], which allows humpback whales to more readily adapt to changes in the abundance and composition of forage species. Responding only to changes in the abundance and distribution of krill, blue whales can adapt to major interannual changes only through prey search effort, not prey switching. In this regard the potential for blue whales to increase efficiency through social foraging, using acoustic communication in collective regional prey searching [93], may represent an essential strategy and an evolutionary adaptation accompanying prey specialization. The efficacy of long-distance acoustic communication interpreted for blue whale social foraging may also represent an evolutionary opportunity for humpback whales, if they can recognize heightened foraging call activity of blue whales and respond to take advantage of ephemeral krill hotspots identified and acoustically signaled by a sympatric mysticete species. Because humpback whales produce sounds in the frequency range of blue whale foraging calls, it is probable that they could hear such heightened call activity from blue whales.

### Energetic variations in ecosystem state

Interannual variations in forage stocks strongly depend on large-scale, highly energetic ocean climatic fluctuations [16,26,77]. Very large interannual variations in forage species occurred during our study period (Fig 7c). Longer records from the central CCE, spanning 23 years, show that krill reached their lowest abundances during year 4 of our study while anchovy rose to their highest abundances during years 4–6 [79]. Such large variations in forage species abundance and composition would naturally affect the behavioral ecology of whales within this essential foraging habitat of the northeastern Pacific. Yet understanding the complex behavioral ecology of such far-ranging predators is difficult, and the powerful propagation of sound produced by highly soniferous species confers a distinct advantage to the use of passive acoustic monitoring. We considered multiple hypotheses to explain the large interannual variations in acoustic detection by integrating diverse methods of sensing whales and their ecosystem. This data intensive effort identifies the consequences of foraging ecology as a significant and persistent driver of interannual variations in the acoustic detection of these whale species. Recognition of this complex relationship, in turn, has important implications for how we interpret passive acoustic monitoring data for scientific and management purposes.
